# Support for the ‘Pets as Ambassadors’ hypothesis in men: Higher animal empathy in Australian pet-owners vs non-owners and farmers

**DOI:** 10.1017/awf.2024.25

**Published:** 2024-05-03

**Authors:** Georgia Anne Frampton, Jessica Lee Oliva

**Affiliations:** 1 Institute for Social Neuroscience Psychology, Melbourne, VIC, Australia; 2 Department of Psychology, College of Healthcare Sciences, James Cook University, Townsville, QLD, Australia

**Keywords:** animal agriculture, animal welfare, cognitive dissonance, contact hypothesis, human-animal interactions, moral disengagement

## Abstract

Human empathy towards non-human animals (Animal Empathy; AE) has shown a strong gender bias, with women demonstrating higher levels than men. This study aimed to investigate the influence of animal experiences on AE in a male-only sample. It was hypothesised that there would be different levels of AE between men with experiences caring for pets, men with experience in animal agriculture, and men with limited animal experiences. Ninety-one Australian men (18yrs+) completed an online survey evaluating their level of AE using the Animal Empathy Scale (AES). Additionally, they were asked what in their experience they think has influenced their beliefs about how animals think and feel. As expected, AE levels differed significantly between groups, with those in the pet ownership experience group demonstrating higher AE levels than the other two groups. All three groups displayed high endorsement for direct interactions with animals in adulthood as being most influential in shaping their beliefs about how animals think and feel. However, our quantitative results support the idea that not all experiences are worth the same, with the responsibility and sacrifice involved in pet caring appearing to be most influential to the development of AE. These findings have implications for the importance of human-animal interactions in understanding animal sentience and the development of AE in males.

## Introduction

There is evidence to suggest that owning animals as pets may act as a stepping-stone to increased Animal Empathy (AE) more broadly (otherwise known as the ‘Pets as Ambassadors’ hypothesis; Serpell & Paul 1994). For example, Paul and Serpell (1993) found an association between childhood pet ownership and more positive attitudes towards non-pet animals. Further, Paul (2000) demonstrated that ownership of a pet both in childhood and adulthood was associated with higher AE than non-ownership. However, Daly and Morton (2009) demonstrated that while pet ownership during childhood has a positive impact on AE, pet ownership during adulthood resulted in greater empathic gains, particularly for those who owned both cats and dogs. In adults, both pet contact and pet attachment have been associated with greater moral concern for a variety of animals, as well as decreased speciesism (Auger & Amiot 2017; Possidónio et al. 2021). Pet ownership has also been associated with greater concern for pig welfare (particularly if the pet was viewed as “a child” or member of the family; Vandersen & Hötzel 2021), and a predictor of positive attitudes towards animals and greater concern for animal welfare (Martens *et al.* 2019). Importantly, female gender was a positive predictor in this sample (Martens *et al.* 2019).

Indeed, studies have typically found gender to be a consistent predictor of empathic behaviours and sentiments, with women displaying higher levels of self-reported empathic attitudes and behaviours towards both humans and non-human animals (Paul 2000; Heleski et al. 2004; Herzog 2007; Colombo *et al.* 2016), as well as greater beliefs in animal sentience (Clarke & Paul 2019) and human-animal continuity (Colombo *et al.* 2016), less support for the use of animals in research (Hagelin *et al.* 2003), and increased concern for animal welfare and rights (Phillips *et al.* 2011), compared with men. Men have also been reported to partake in more frequent behaviours that adversely affect animals than do women (e.g. hunting and animal abuse behaviours including neglect, bestiality, and involvement in animal fighting rings) (Herzog 2007). Lack of AE has also been associated with interpersonal aggression in men. For example, Febres *et al.* (2014) found that animal abuse behaviours were present in almost half of all male perpetrators of intimate partner violence in a US sample. In addition, Flynn and Graham (2010) found that up to 75% of pet-owning female victims of intimate partner violence reported that their pets were threatened or harmed by their male partner. This highlights how compromised abilities to feel empathy in men can have devastating effects on both animals and humans alike. Thus, increasing our understanding of the role human-animal interactions play in the development of AE has far-reaching implications, particularly in males.

Two of the most popular self-report measures assessing human perceptions of the internal experiences of non-human animals and how they are treated include: the Animal Attitude Scale (AAS; Herzog *et al.* 1991; Herzog 2007), and the Animal Empathy Scale (AES; Paul 2000). The majority of AAS items pertain to wild animals or animals used for utilitarian purposes. That is, for purposes that ‘benefit’ humans (i.e. farming, research, entertainment, profit). The exception is one question about attitudes towards the breeding of purebred dogs over shelter adoption (Herzog *et al.* 1991; Herzog 2007). The AES is more balanced with roughly half of the items pertaining to pet animals and the other half relating to animals on television, animals ‘in general’, and caged animals, e.g. birds, animals in zoos etc. (Paul 2000). However, an overarching construct of AE is a potentially problematic one, because not all animals are perceived as equal. For example, findings from Taylor and Signal (2009) reveal that there are important differences in attitudes towards the treatment of animals when the animal serves a utilitarian purpose for humans, compared to when they do not (i.e. pest animals). When it comes to animals that possess their own intrinsic value (i.e. our pets), attitudes towards their treatment are different again, with more pro-animal attitudes directed towards pets, followed by profit animals, then pest animals (Taylor & Signal 2009).

While we may hold our pets in higher regard than other animals, our relationship with them may allow them to take on an ‘ambassador’ role for all animals by increasing AE more generally (Serpell & Paul 1994). Similarly, experience and knowledge gained through working with animals in the agricultural industry may help to promote empathy in farmers, as it is through this experience that farmers learn about animal behaviour and cognition, supporting the so-called “contact hypothesis” (Allport 1954). However, it may also act as a barrier to developing empathy, given that the animals they would develop empathy for would inevitably be exposed to farming practices that may cause them sufferance and death. To protect themselves from this pain they may therefore discredit the internal experience of the animals so as to be able to do their jobs emotionally unharmed, i.e. by morally disengaging to avoid feelings of cognitive dissonance (Gradidge et al. 2021). This has been demonstrated in veterinary students with Colombo *et al.* (2016) demonstrating that AE declines over time in this population, which may be a protective mechanism enabling them to remain on a career path with the potential to be highly emotionally challenging. Reduced AE in veterinary students could also reflect role-modelling within the industry, for example, veterinary medicine has its roots as a masculine profession and may still glorify stereotypically masculine attitudes and behaviours, for example, requiring “brutal and inhumane” treatments and “unsentimental” practitioners (Irvine & Vermilya 2010; p 59). Similar sentiments are also seen in the agricultural industry. For example, a thematic analysis on data from a semi-systematic review of factors that influence farmers’ views on farm animal welfare, found that animal handling practices were often purely carried out on a collective tradition of “the way things are” (Balzani & Hanlon 2020; p 12). However, there may be some merit in adopting more empathic approaches to farming practices, for example, Hanna and colleagues (2009) found that a higher milk yield in dairy cows was positively correlated with higher empathy levels of farmers, and Hemsworth *et al.* (2002) demonstrated that a cognitive-behaviour intervention designed to improve the attitudes and behaviours of stockpeople towards their cows could improve cow productivity. More positive farmer-animal relationships and pro-animal attitudes have also been associated with higher empathy and better productivity in pig farming (Kauppinen *et al.* 2012; Jääskeläinen et al. 2014; Pol *et al.* 2021).

It is important to understand the factors that contribute to the development of AE as this has benefits not only to the animals themselves, but to their productivity in an agricultural sense, and may also help foster empathy in interpersonal contexts. Research has demonstrated that experience with both pet and farm animals may be important, however, it is unclear whether all experiences are worth the same. This is particularly important to investigate in a sample of men, as men typically demonstrate lower levels of empathy compared to women. Additionally, women have recently dominated the file of studies concerning human-animal relationships, with the lack of male representation compromising the validity of studies (Herzog 2021). Hence, this study will aim to: (i) investigate the influence experience with animals has on AE in an Australian sample of men; and (ii) capture the constructs that underpin male beliefs and perceptions of animal sentience. It is hypothesised that there will be differing levels of AE between participants in the following three groups: (i) participants with personal experience as the primary owner of a companion (i.e. pet) animal but not with animals in the agricultural industry; (ii) participants with personal experience working with animals in the agricultural industry (with or without pet experiences); and (iii) participants who have limited experience with animals.

## Materials and methods

### Participants

To be included in the study, participants were required to be Australian citizens, male, 18+ years old, and who self-identify as belonging to one of the following three ‘animal experience’ groups: (i) ‘*I have personal experience as the primary owner/main caregiver of a companion (i.e. pet) animal*’ – participants self-selected into this group if they were the current or past owner of a pet, and did not have experience working with animals in the agriculture industry; (ii) ‘*I have personal experience working with animals in the agricultural industry’* – participants could self-select into this group if they worked in the animal agricultural industry (with or without pet experiences); or (iii) ‘*I have limited experience with animals’* – participants could self-select into this group if they had never been the primary owner of a pet and did not have experience working with animals in the agricultural industry. Individuals not identifying as male were excluded from the study.

### Survey

A survey was administered to participants via the online platform Qualtrics (Provo, UT, USA). The order of administration was the same for each participant; they were first given a series of demographic questions including their age, gender, education, occupation, living arrangements and parental histories, they were then asked about their animal experience for grouping purposes, administered the Animal Empathy Scale (AES; Paul 2000), and finally asked to provide a free-hand response to the following question: *‘What in your experience do you think has influenced your beliefs about how animals think and feel?’*

### Animal Empathy Scale (AES; Paul 2000)

The AES contains 22 statements rated on a nine-point Likert scale which explores empathetic attitudes towards animals. Respondents are asked to rate their level of agreement or disagreement from ‘agree very strongly’ (1) to ‘disagree very strongly’ (9). Eleven questions are negatively scored (e.g. ‘*It upsets me to see animals being chased and killed by lions in wildlife programs on TV’*) and eleven questions are positively scored (e.g. *‘I get annoyed by dogs that howl and bark when they are left alone’*) such that higher scores reflect higher empathy. Total scores are derived by summing the scores of all 22 statements together. Thus, total scores can range from 22 (minimum) to 198 (maximum). Previous studies have shown the AES to have good internal consistency (α = 0.78; Paul 2000, α = 0.83; Colombo *et al.* 2016). In the current study, Cronbach α = 0.88.

### Ethical approval

The study received ethics approval from the Institute of Social Neuroscience Ethics Committee (HREC Reference number: 201201).

### Procedure

Promotion of the study occurred via online advertisements on social media (i.e. Facebook and Twitter), and through sharing of the advertisement in the personal and professional networks of the researchers. Data collection occurred over a period of six weeks (9 February–25 March 2021), wherein participants used their own personal devices to click on a hyperlink to access the secure web-based platform, Qualtrics, where the survey was hosted. Participation was completely voluntary, and neither payment nor incentive was offered to participants for completion. Participant consent was gained after participants read the plain language statement upon opening the link and ticked ‘*Yes, I am aged 18 years or over and I want to participate in the study’.* Participants could withdraw at any time by exiting the survey. The survey took approximately 10 min to complete.

## Results

### Quantitative analysis

Raw data were exported from Qualtrics to Statistical Package for the Social Sciences (SPSS Version 26.0; IBM Corp® 2019). The dataset was examined for missing data, two missing data-points were observed for the ‘occupation’ variable (one from the pet owner group and one from the limited experience group) and left as such.

### Description of the sample

A total of 91 participants completed the study (41 primary owners of companion animals, 28 with experience in the animal agricultural industry, and 22 with limited animal experience). Of the participants in the group with experience as primary owners of companion animals (currently or in the past), 38 owned dogs, 21 owned cats, and eleven owned other animals, commonly: birds, fish, turtles, rabbits and guinea pigs, with one person who owned chickens and goats, and one who owned snakes. Of the participants in the group with experience in the animal agriculture industry, 26 also owned (currently or in the past) a non-working pet animal, with mostly birds or fish reported, and 21 owned a working animal. All of these were dogs (mostly sheep or cattle dogs), with the exception of one participant who reportedly kept working horses. The majority of participants in the agricultural experience group had worked with more than one species with 17 who had worked with beef cattle, 14 with chickens, 15 with sheep, ten with dairy cows, nine with pigs, three with goats, and one with alpacas. See Table 1 for a complete summary of participant descriptive frequencies.

**Table 1.** Demographic data of men (n = 91) participating in the study grouped according to their experience with animals

	Group		
	Experience as primary companion animal owner (n = 41)	Experience with animals in the agricultural industry (n = 28)	Limited experience with animals (n = 22)
**State/Territory of residence**			
Victoria	26	16	19
Western Australia	1	2	1
South Australia	1	3	1
New South Wales	5	3	1
Queensland	5	4	0
Australian Capital Territory	3	0	0
**Rural or Urban area of residence**			
Rural	7	21	1
Urban	31	7	21
Unsure	3	0	0
**Highest Education Level**			
Year 10 or below	1	0	0
Year 11/12	3	3	1
TAFE course or trade apprenticeship	14	7	1
Undergraduate degree	8	12	7
Postgraduate degree	15	6	13
**Current occupation**			
Student	2	3	3
Retired/unemployed	6	3	1
Labourers/admin/service workers	11	2	3
Academics/Professionals/Managers	20	2	14
Work with animals	1	18	0
Did not disclose	1	0	1
**Parental Status**			
Parent of biological child/children	17	16	7
Parent of adopted child/children	1	0	0
Never had children	21	12	14
Other	2	0	0
Prefer not to say	0	0	1

### Differences between groups

The presence of extreme outliers was checked using an assessment of z scores ≥ ± 3.29, and a visual inspection of boxplots, with one found in the agricultural experience group (an extreme low AES score of 48). Extreme outliers have the potential to be overly influential on the ANOVA model, therefore it was decided to winsorise the data-point (i.e. to replace it with the next extreme score in the agricultural experience group, that was not an outlier – thus the AES score was changed from 48 to 101). In addition to this, we ran the same analysis on the original and winsorised datasets. While the results were unchanged, the winsorised dataset resulted in less error. Therefore, all further data analysis was conducted using the winsorised dataset. Descriptive Statistics for the AES calculated using the windsorised dataset can be seen in Table 2.

**Table 2.** Mean (± SD) and range of animal empathy scores of men according to their experience with animals

Group	Mean	± SD	Range
Experience as a primary companion animal owner (n = 41)	153.71	20.70	104–198
Experience with animals in the agricultural industry (n = 28)	136.18	20.73	101–167
Limited experience with animals (n = 22)	126.59	22.02	87–168

n = 91

Homogeneity of variances was achieved, as assessed by Levene’s test for equity of variances (*P* = 0.978). Visual inspection of histograms and P-P plots confirmed that the data were normally distributed. As such, a one-way ANOVA was conducted to investigate if there was a difference in AES scores across the three groups. The ANOVA indicated that there was a significant difference in empathy across the three experience type groups, *F*_2, 88_ = 13.32; *P* < 0.001, η2 = 0.232. Tukey’s *post hoc* analysis revealed the companion animal owner group demonstrated significantly higher AES scores as compared to the limited experience group, with a large effect (27.12, 95% CI [13.86, 40.37]) (*P* = 0.001, *d* = 1.04), and the companion animal owner group as compared to the agricultural group, with a medium-large effect (17.53, 95% CI [5.24, 29.82]) (*P* = 0.003, *d* = 0.72). No difference was found between the agriculture group and the limited experience group (9.59, 95% CI [4.70, 23.87] (*P* = 0.251).

### Qualitative thematic content analysis

The researchers independently identified themes from individual responses to the question ‘*What in your experience do you think has influenced your beliefs about how animals think and feel?’* in blocks of ten, before coming together to discuss individual findings and form a consensus for theme names, definitions, and response categorisation. This process continued in an iterative manner until all responses were analysed. Three participants (two from the agricultural experience group and one from the limited experience group) left this question blank, and one participant from the pet ownership experience group responded with “*not sure*”. Additionally, two participants from the pet ownership experience group and one from the agricultural experience group provided responses that did not address the question. As such, these cases were removed from the sample size when calculating frequency endorsements. Themes endorsed by participants from all experience groups can be seen in Table 3.

**Table 3.** Frequency of responses (sorted into themes) to the question ‘*What in your experience do you think has influenced your beliefs about how animals think and feel?*’ by men (n = 84) according to their experience with animals

		Group Frequencies and Percentages		
Theme	Example quotes	Primary companion animal owners (n = 38)	Those with experience in the animal agriculture industry (n = 25)	Those with limited experience with animals (n = 21)
First–hand experiences through work/livelihood	*Working in neuroscience* *Working in shelters* *Using animals as assets or as working animals*	2 (5.3%)	15 (60.0%)	1 (4.8%)
First–hand animal ownership experiences	*Having the responsibility of my dog formed a really close bond* *Being a cat ‘dad’ – I recognise their moods and idiosyncrasies! They are so individual!*	21 (55.3%)	8 (32.0%)	0 (0%)
First–hand experiences with–, and knowledge of–, other’s pets	*Knowing friends with animals and how they explain pet behaviour to me, and what pets mean to them* *Through outings with friends over the years, and meeting their pets and seeing how they interact*	1 (2.6%)	1 (4.0%)	10 (47.6%)
First–hand experiences throughout childhood	*I had pets as a child* *Growing up on a farm*	10 (26.3%)	4 (16.0%)	4 (19.0%)
Knowledge of animal biology, sentience, and/or animal cognition	*Being aware that animals are sentient beings* *Capacity for pain/distress perception*	8 (21.1%)	3 (12.0%)	5 (23.8%)
Values and beliefs relevant to animal welfare/ethics	*Parents upbringing. Reincarnation.* *Having a mother from the UK where companion animals are traditionally treated better than in Australia and having a father from country Victoria who had very different ‘country’ attitudes to animals – i.e. dogs should be kept outside*	6 (15.8%)	3 (12.0%)	2 (9.5%)
Media	*Television and YouTube documentaries* *Watching wildlife programmes on television, particularly the magnificent programmes of Sir David Attenborough*	3 (7.9%)	1 (4.0%)	3 (14.3%)

Note: Individuals could only endorse a theme once, but could endorse more than one theme.

Frequencies were calculated by summing the total number of participants who endorsed a theme. Percentages were calculated by dividing the frequency by the group total.

As can be seen from Table 3, first-hand experiences with animals were the single most important factor in forging participant beliefs regarding what animals think and feel. However, these first-hand experiences differed in nature across the three groups, with the agricultural experience group endorsing their experiences through work/livelihood the most, the companion animal experience group endorsing their personal ownership experiences the most, and the limited experience group endorsing their experiences with other people’s pets the most. This was closely followed by first-hand experiences throughout childhood (slightly higher in the ownership group), as well as knowledge of animal biology, sentience and cognition (slightly lower in the agricultural group). While less commonly endorsed overall, values and beliefs around animal welfare and ethics were more influential for the ownership and agricultural experience groups, while media influences were more commonly endorsed in the limited experience group.

Three participants from the limited experience group provided unique ideas that could not load onto the main themes presented in the table. These responses included, “*Zoo visits”, “I had stray cats as pets”, and “Seeing them out in public”.* Similarly, the pet ownership experience group included three unique responses, “*Observing wild animals”, “Life experience in general”,* and *“I’m also not a sociopath so that helps”.* Finally, the agricultural experience group included one unique response, “*A feeling they are friends of mine, and I should look after them as best I can”.* Participants with unique responses were included in sample size calculations in Table 3.

## Discussion

The current study aimed to quantify AE in three sub-samples of Australian males: pet owners, non-owners, and farmers, and to identify factors that contribute to their beliefs and perceptions of animal sentience. Results support the hypothesis that participants with different types of animal experiences have differing levels of AE. Empathy levels were significantly higher in the companion animal (i.e. pet) ownership group when compared to both the limited experience and agricultural experience groups (with large and medium-large effects, respectively). These findings are consistent with previous studies concerning the effect caring for a pet has on empathic development towards animals (Paul 2000; Daly & Morton 2009; Auger & Amiot 2017; Martens *et al.* 2019; Possidónio et al. 2021; Vandersen & Hötzel 2021). Qualitative insights from the current study do suggest that first-hand pet ownership experiences may be an important factor explaining why this group possessed significantly higher empathy levels than the other two groups (see Table 3), providing support for the ‘Pets as Ambassadors’ hypothesis (Serpell & Paul 1994), but only if the pet owner is not also a farmer.

The vast majority of men in the agricultural experience group were also owners of working and non-working animals (see sample description). The reduced levels of AE demonstrated in this group might be explained by differences in pet type. For example, while most pet owners owned dogs and cats, most farmers owned pet birds and fish. However, most of their working animals were dogs, and it is possible that these dogs were considered to have a dual pet role as well. Despite possessing pet ownership experiences, it is interesting that farmers most frequently endorsed their first-hand experiences through work/livelihood as being most influential in their thoughts and perceptions as to how animals think and feel. There are a couple of explanations for this, the first being that there may be something more to be gained or understood from working closely with non-domesticated farm animals in terms of understanding how animals think and feel. However, for this to be true we would need to accept that greater understanding about animal sentience does not necessarily correlate with higher levels of AE, as our findings demonstrated that the men with agricultural experience possessed significantly lower levels of animal-directed empathy than men with experience caring only for companion animals. Moreover, ‘*knowledge of animal biology, sentience, and/or animal cognition*’ was endorsed least by this group (see Table 3), suggesting that they rely more upon their own personal experiences than ‘textbook’ facts about animals. This is interesting as the majority were university educated, as per the other two groups (see Table 1). Alternatively, due to the nature of their work, men with agricultural experience may view their relationships with all animals, including pets, differently to those who only own pets. For example, a ‘pet’ for a farmer might be a domestic animal that also performs work duties and therefore lies somewhere between Taylor and Signal’s (2009) “pet” and “profit” categories, with their farm animals sitting firmly in the “profit” category. An Australian survey found that while 65% of people considered the welfare of domestic pets to be most important, only 49.3% said the welfare of farm animals was paramount, and 41% of participants said the welfare of animals, in general, was essential (Coleman *et al.* 2015). This supports the idea that one’s attitudes towards animals as sentient beings with an ability to suffer and experience emotions can vary relative to the species in question. In contrast, many pet owners who are not also farmers view their pets as family members (McConnell *et al.* 2016), and as Vandersen and Hötzel (2021) found, considering pets as family members positively predicts empathy towards farm animals.

Cognitive dissonance and moral disengagement might explain why farmers may not view their animals as family members (Gradidge et al. 2021). For example, production practices may lead to animal suffering and death which would be difficult for farmers to inflict upon members of their family. By distancing themselves emotionally from their animals, they also distance themselves from the emotional pain they would feel for them. Our current findings do suggest that this group relies less than the other two groups on ‘*knowledge of animal biology, sentience, and/or animal cognition*’ in their perceptions and beliefs about what animals think and feel. Thus, they may also ascribe farm animals a lesser ability to suffer than other animals (i.e. domesticated pets) or humans, thereby reducing the mental tension that comes from feeling empathy towards their animals and being responsible for them. However, a previous study investigating this in pig farmers was not able to support this notion (Peden *et al.* 2020), and although this theme was endorsed least by this group, select qualitative insights from farmers in the current study reveal that at least some of them are aware of the internal experience of their animals:

*“They have feelings, emotions, pain, loss, grief…”*

Our finding that farmers demonstrate lower levels of empathy compared to those who only have pets is consistent with previous studies concerning those who use animals as part of their livelihood outside of the agricultural industry. For example, veterinary students are more likely to rate different castration techniques carried out without anaesthetic, the withholding of food from a healthy animal, and the force-feeding of a healthy animal, as humane if they had aspirations to work with food animals, as compared to small animals (Levine *et al.* 2005). Importantly, veterinary medicine is extremely gender segregated with nearly all the students in large animal tracks male (Irvine & Vermilya 2010). Veterinary students have also been shown to hold decreasing levels of empathic sentiments for animals according to years of study, and this was more pronounced in males compared to females (Colombo *et al.* 2016).

In line with results from Daly and Morton (2009), pet experiences in adulthood appear to be more influential than pet experiences in childhood. This may come down to the difference between being personally responsible for a pet, as compared to simply interacting with and being close to a pet (as in childhood). While children may be allocated certain caring responsibilities for the family pet, such as feeding it, cleaning up after it, or in the case of dogs, walking it (Muldoon *et al.* 2015), larger decisions about the health and welfare of the pet, as well as veterinary and end-of-life decisions, are usually left to adults. Children most likely will not have experienced or had the responsibility of carrying such burdens, and so their experiences may be likened to those men in the limited experience group, who endorsed first-hand experience with other people’s pets as the most important for development of beliefs and perceptions of what animals think and feel. Larger decisions associated with high levels of responsibility may prime neural and endocrine systems that increase the capacity for empathic concern, as has been observed in parents of newborns (Gómez-Carvajal *et al.* 2020). Previous studies have shown parental history to positively predict the belief in the empathic ability of pet dogs to ‘pick up’ on their owner’s emotions (Oliva *et al.* 2016). However, parental history does not appear to have affected our findings relating to human empathy towards animals, as the highest percentage of parents was found in the farmer group at 57% (compared with 44% of pet owners and 32% of men with limited animal experiences). There are also no guarantees that a family pet will have any impact on children at all, research suggests that it depends upon the bond the child shares with the animal. For example, a study conducted by Poresky and Hendrix (1990) found that family pets positively influenced social competency and empathy development in children if the child was bonded with the animal, as opposed to the animal merely being ‘present’ in the household. In their review, Beetz *et al.* (2012) summarise the support for the role of oxytocin in this bond, which has been associated with empathy development (Connor *et al.* 2018). Connor *et al.*’s findings support that the oxytocin receptor plays a role in AE in humans, however their analysis was not carried out separately for men and women.

Pet ownership is associated with a variety of sacrifices made by the owner without the expectation of financial remuneration (this contrasts with farmers who may make sacrifices in order to gain a profit). However, there is evidence to suggest that these sacrifices only enhance the owner-pet relationship, rather than impair it. For example, Freeman *et al.* (2020) found that fifty percent of owners of dogs with congenital heart disease reported negative impacts on their quality of life. Yet despite the hardships (e.g. difficulties medicating and caring for a dog with chronic illness, worry associated with their dog’s worsening condition, fear over dog loss etc), no owners reported a weakened relationship because of this, and in fact, the majority revealed that the process of caring for their dog only caused their relationship to become stronger. Similar thought processes are evident in pet owner responses in the current study, for example:

*“Having the responsibility of my dog formed a close bond.”*

Taken together, these findings support the idea that a history of responsibility and sacrifice for a personal pet, without the expectation of financial remuneration, can open one up to the possibility of an enhanced pet-owner bond which, in turn, can increase one’s capacity for AE in general.

### Strengths, limitations, and future directions

A major strength of the current study is the use of a sample of only men. This allowed for the complete control of the bias females often bring to studies concerning human-animal interactions and animal-directed empathy (Herzog 2021). However, further research should consider the limitations of sampling just males, as they have proven to be particularly difficult to recruit. While the original intention was to have groups that were mutually exclusive in experience type, we found achieving this to be almost impossible. This is particularly evident in the agricultural experience group of whom most were also owners of working and non-working domestic animals, as well as the limited experience group, with most participants having had some experiences and interactions with animals. Further, as most of the non-working animals owned by farmers were limited to birds and fish, the ambassadorship of ‘pets’ may be limited to certain species, like the non-working cats and dogs most commonly reported in the pet ownership group. Future research could also look at whether ambassadorship is related to the degree of interaction the owner has with their pet. Further research might also consider the possibility of assessing what influence owning working animals, simultaneously with companion and profit animals, has on a farmer’s AE and whether this differs according to the animal in question. Additionally, further research might consider adding additional experience-type groups, for example, groups that encompass different professions that use or involve animals, while being mindful of minimising potential crossover of experiences. It is important to keep in mind that while the qualitative results do suggest that AE is enhanced through pet ownership, due to the cross-sectional nature of this study, it is impossible to know whether men with higher AE are more drawn to become pet owners than men with lower AE. In other words, that the AE level is influencing whether one becomes a pet owner, rather than *vice versa.* Longitudinal studies that look at AE development over time (i.e. before and after pet acquisition) would be able to address this.

### Animal welfare implications

The current findings suggest that animal interactions play an important role in the development of animal-directed empathy in men. This has direct implications for animal welfare as the quality of an animal’s future interactions with men will be dependent upon the man’s past interactions. However, not all experiences are equal, and experiences that involve responsibility and sacrifice without the expectation of financial remuneration, such as those made in the context of an adult owner-pet relationship, appear to be most influential in the development of generalised empathy towards animals. This supports the idea that caring for pets might help men to understand the experience of all animals through the eyes of their beloved pet, allowing their pet to act as an ‘ambassador’ for animals more generally. Qualitative findings suggest that first-hand animal interactions in any context (i.e. through work, ownership, or other people’s pets) help to shape men’s beliefs about how animals think and feel. Being able to appreciate animals’ sentient nature is the first step towards ensuring that their welfare is considered, therefore, animal interactions should be maximised for any man responsible for an animal’s welfare. Knowledge of phenomena that may stunt the development of AE, such as cognitive dissonance and moral disengagement, should also be maximised. The study’s findings are likely to also extend to women, however, the act of caring for a pet may be more important in empathy development in men, who typically demonstrate lower levels compared to women. Pet caring may also result in a reduction of behaviours more commonly engaged in by men that negatively impact an animal’s welfare, such as hunting, animal abuse, and the support of animal fighting. It is important to note, however, that researchers have suggested that the ‘ownership’ of an animal, by its very nature, infers an unequal relationship, whereby the pet lacks certain freedoms and choice (Hockenhull & Furtado 2021; Oliva & Green 2021). Therefore, it could be considered quite the paradox that, in order to achieve high levels of AE one must ‘own’ a pet, yet at the same time ownership of a pet strips the animal of basic rights and freedoms that should arguably invoke empathic sentiments.

Finally, we would like to stress that the decision to acquire a companion animal should always be well thought out with a commitment to care for the animal for the duration of its life and find ways to enhance its welfare throughout this time. For those not in the position to care for an animal, finding other means to interact with animals (e.g. through family or friends), appears to offer some benefit. While it may not be possible to avoid the cognitive dissonance that comes with farming practices, training for such roles should include awareness and understanding of such phenomena so that minimisation of negative internal animal experiences such as pain and distress are reduced in these roles.

## Conclusion

In conclusion, this study revealed that men with experience only as companion animal owners (and not in the agricultural industry) possess higher levels of animal-directed empathy as compared to men with experience in the agricultural industry and men with limited animal experiences, supporting the ‘Pets as Ambassadors’ hypothesis. Qualitative findings suggest that first-hand animal experiences are key in shaping beliefs and perceptions about what animals think and feel, however, the most frequently endorsed type of first-hand experience differed by group. Potential explanations for these findings are discussed, including the role of responsibility and sacrifice in the development of empathy in pet owners, and the potential role of cognitive dissonance and moral disengagement in stunting empathic sentiments in men who rely upon animals for work.
